# Simple platform for chronic imaging of hippocampal activity during spontaneous behaviour in an awake mouse

**DOI:** 10.1038/srep43388

**Published:** 2017-02-27

**Authors:** Vincent Villette, Mathieu Levesque, Amine Miled, Benoit Gosselin, Lisa Topolnik

**Affiliations:** 1 Neuroscience Axis, CHU de Québec Research Center (CHUL), Laval University, Québec, PQ, G1V 4G2, Canada; 2 Department of Biochemistry, Microbiology and Bio-informatics, Laval University, Québec, PQ, G1V 0A6, Canada; 3 Department of Electrical and Computer Engineering, Laval University, Québec, PQ, G1V 0A6, Canada

## Abstract

Chronic electrophysiological recordings of neuronal activity combined with two-photon Ca^2+^ imaging give access to high resolution and cellular specificity. In addition, awake drug-free experimentation is required for investigating the physiological mechanisms that operate in the brain. Here, we developed a simple head fixation platform, which allows simultaneous chronic imaging and electrophysiological recordings to be obtained from the hippocampus of awake mice. We performed quantitative analyses of spontaneous animal behaviour, the associated network states and the cellular activities in the dorsal hippocampus as well as estimated the brain stability limits to image dendritic processes and individual axonal boutons. Ca^2+^ imaging recordings revealed a relatively stereotyped hippocampal activity despite a high inter-animal and inter-day variability in the mouse behavior. In addition to quiet state and locomotion behavioural patterns, the platform allowed the reliable detection of walking steps and fine speed variations. The brain motion during locomotion was limited to ~1.8 μm, thus allowing for imaging of small sub-cellular structures to be performed in parallel with recordings of network and behavioural states. This simple device extends the drug-free experimentation *in vivo,* enabling high-stability optophysiological experiments with single-bouton resolution in the mouse awake brain.

Neurons are embedded in anatomical and functional circuits to form highly dynamic computational clusters in various brain states. The brain states are related to the ongoing behaviour of the experimental subject but they are altered under anaesthetized conditions^1^,^2^,^3^. The cell activities in drug-free mammals during behaviours were first recorded more than 50 years ago thanks to the development of wire recordings^4^. At present, due to methodological progress, the use of optrodes in rodents targeted with genetic or viral approaches facilitates reliable recordings of specific cell types in different behavioural and brain states^5^. In addition, two-photon imaging technique provides a complementary approach with several advantages, including high spatial cellular resolution^6^,^7^, topographical neuronal mapping^8^, multicolour genetically based cell identification^9^,^10^, and simultaneous large-population imaging^11^,^12^.

Conventional two-photon microscopy requires a stable microscopy base, which makes freely moving behavioural experiments challenging. To address this issue, several variable geometry treadmills have been designed, including spherical^13^,^14^,^15^, cylindrical^16^, flat air-lifted^17^, and linear types^18^,^19^,^20^, which allow a mouse to move while the head is fixed under a microscope’s objective lens. From an experimental viewpoint, the head fixation procedure is convenient, although it requires an extended animal habituation to the experimental apparatus. In addition to spontaneous behaviour^12^,^14^,^20^,^21^, different learning paradigms can be implemented in head-fixed animals^13^,^15^,^19^,^22^,^23^,^24^. While a typical experiment begins from repetitive animal handling and training, little is known about the inter-animal vs inter-day variability in spontaneous behaviour of head-fixed mice, and the evolution in neuronal activity patterns in relation to evolving behaviour, which is important for data validity and reproducibility.

Here, we developed a head fixation platform that is easy to implement within a conventional two-photon imaging system for chronic Ca^2+^ imaging to be performed in parallel with recordings of hippocampal oscillations. We took advantage of the hippocampal CA1 area as a model because its cellular and network activities have been characterized extensively in freely behaving rodents^25^,^26^,^27^,^28^,^29^,^30^,^31^. Moreover, there is a recognized precise relationship between the CA1 activity and animal speed^32^,^33^. In addition to well-defined behavioural patterns consisting of immobility and locomotion, the head fixation platform developed here allows to analyse the animal habituation rate and to count walking steps. Furthermore, it enables high-precision analysis of Ca^2+^ signals in single neurons, neuronal dendrites and individual axonal boutons with a superior stability in locomoting mice. We used the platform to analyse the individual, inter-session and intra-session variability in animal behaviour as well as cell-to-network recruitment. Our data reveal the stereotyped activity patterns of CA1 neurons in parallel with variability in motor activity.

## Results

### Head fixation platform design

**Figure 1:**
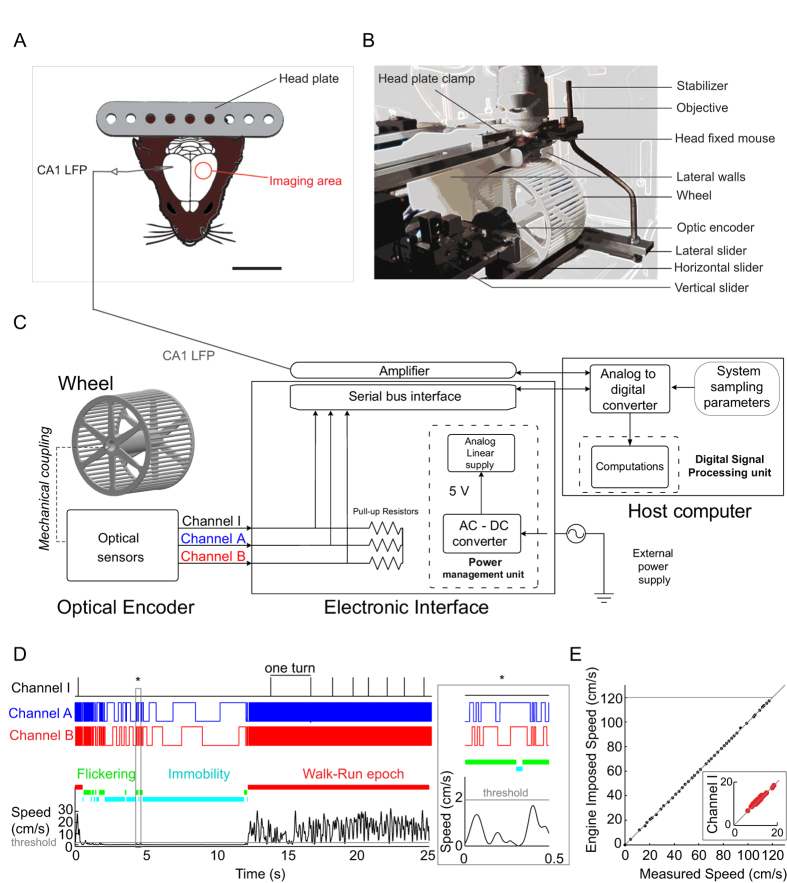
Experimental set-up. (**A**) Schematic showing a mouse’s head equipped with a head plate and the LFP recording CA1 electrode. The location of the imaging area is indicated (red). Scale bar represents 10 mm. (**B**) Schematic showed a head-fixed mouse on the experimental device. The objective on top of the mouse’s head was used for calcium imaging. (**C**) Design of the optical encoder electronic circuit with three output BNC channels labelled as channel I (black), channel A (red), and channel B (blue), component maps for the optical encoder and power plug connector, power plug circuit, pull-up resistances, and output BNC. (**D**) Representative traces from the three channels when the mouse exhibited spontaneous behaviour. Channel I (black) indicates a full wheel rotation (one turn = 24.19 cm) between two 5-V inflections. Channels A (red) and B (blue) indicate fringes (each square signal is equal to 1/500 of a turn). Note that these channels were phase-shifted to monitor the direction of the wheel’s rotation. The corresponding instantaneous speed trace (black) and underlying behaviour phases are shown (bottom). The mobility threshold (grey line, 2 cm s^−1^) was used to distinguish run epochs (red bars) from flickering (green bars) and immobility (cyan bars) periods. Grey outline box (*) indicates the zoomed view (right panel, 500-ms duration). (**E**) Scatter plot showing the speed measurements obtained after processing the data from channels (A and B) using the algorithm (see Methods), and the speed imposed by the wheel rotation engine (black dots, Pearson’s correlation coefficient: r = 0.99, p < 0.0001). It should be noted that the linear relationship is shown for the full tested range (0 to 120 cm s^−1^). The insert indicates the real mouse speed (red dots) obtained from the median speed for each turn (from the channel I calculation, see Methods).

Our design strategy was based on the classical head fixation systems where a head plate is implanted over the animal’s head (Fig. 1a, Supplementary Fig. 1). This head plate was fixed twice at its extremities so the animal’s head has a high degree of mechanical stability (Fig. 1a,b). Using this method, there was negligible motion when the animal was immobile but motion can become a limiting factor when the animal runs. To minimize this motion induced by locomotion, we developed a shock absorber-free wheel with three key components: (1) a spring-controlled floating structure, (2) a minimal friction rotating wheel, and (3) a custom-designed soft wheel (Supplementary Fig. 1e). The combination of these three components allowed the construction of a free rotating wheel, which could absorb the motions induced by the limbs and subsequently minimize any brain motion artifacts, although the latter remains to be tested with and without the shock-absorbing mechanism. The wheel was not controlled by any engine, so the mouse could walk and run forwards and backwards without restriction (Fig. 1d). To track the changes in position, speed, acceleration, and deceleration, an optical encoder was fixed on the rotating axis (Fig. 1b,c, Supplementary Fig. 1e). The wheel was assembled with two lateral walls that mimicked the closed arms of typical behavioural mazes (Fig. 1b, Supplementary Fig. 1b). This head fixation device could be installed under a two-photon microscope on any XY-motor controlled platform provided that the space between the platform and the objective exceeds 14 cm (Fig. 1b, Supplementary Fig. 1a). The optical encoder was supplied with power (Fig. 1c) and the analog signals were digitized to reliably track the instantaneous speed at up to 120 cm s^−1^ with millisecond precision (Fig. 1d,e). In summary, we developed a simple experimental platform that is capable of simultaneous electrophysiological recordings and two-photon Ca^2+^ imaging in awake mice *in vivo*.

### Spontaneous behaviour and hippocampal network states

**Figure 2:**
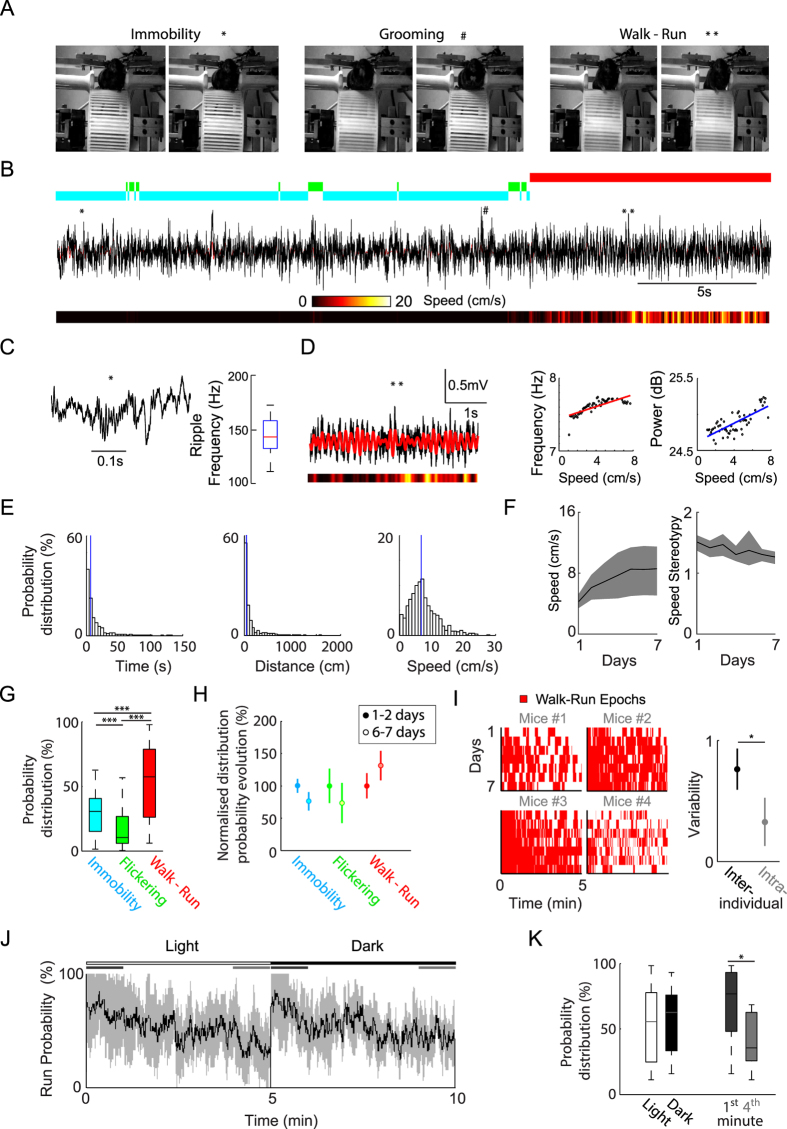
Spontaneous behaviour and hippocampal network states. (**A**) Illustrations of spontaneous behaviour by a mouse, with periods of immobility (left), grooming (middle), and walking-running behaviour (right). (**B**) CA1 LFP recording (middle) and the animal speed (heat map) over a 30-s period. (**C**) Representative ripple event occurring during immobility (*from **B**, left) and graph of the ripple frequency (right). (**D**) Raw (black) and filtered (red) theta oscillations recorded during a locomotion epoch (**from **B**), and a heat map representing the animal speed (left). The frequency-speed (middle, Pearson’s correlation coefficient: r = 0.776, *P* < 0.05) and power-speed (right, r = 0.755, *P* < 0.05) relationships are plotted together with their linear fit. (**E**) Probability distributions of duration, distance, and speed for run epochs (n = 915, 70 sessions, five mice). The median is indicated by a blue line. (**F**) Graphs of the median speed and speed stereotypy over days (mean ± standard deviation, n = 5 mice, bootstrap, *P* < 0.05). (**G**) Graphs showing the distributions of behavioural phases in 70 5-min sessions (cyan: immobility; green: flickering; red: running epochs). ***P < 0.001 (Wilcoxon test). (**H**) Evolution of spontaneous behaviour between the two first and last days (filled and open circles, respectively) (data are expressed in percentage of the first days as mean +/− sem, n = 5 mice). (**I**) Illustration of the run epoch pattern diversity in four mice where the red areas correspond to run epochs (left). Inter-individual (black) and intra-individual (gray) variabilities are shown at right (n = 5 mice, n = 7 days, *p < 0.05, Wilcoxon test). (**J**) Quantification (mean ± standard deviation, n = 5 mice) of the walk-run epoch probability in two 5-min consecutive experimental conditions: light (white) and darkness (black). The first and fourth minutes are labelled with dark and light grey bars, respectively. K. The probability distributions of run epochs in light (white) and dark (black) conditions (left, Wilcoxon test, *P* > 0.5) and during the first (dark grey) or fourth minute (light grey) of the experimental session (n = 10 sessions, five mice, Wilcoxon test, *P* < 0.05).

The mouse was handled according to a progressive scheme. Stable data could be acquired as early as the third day of handling during spontaneous immobility episodes and locomotor activities (walk/run epochs) (Figs 1d and 2a,b). To obtain information about the network states during different behavioural patterns, we recorded the hippocampal CA1 local field potential from the contralateral to the imaging window hemisphere (Fig. 2b). The immobile state including grooming periods was associated with a large irregular activity (LIA) and periodic high-frequency ripple oscillations (median frequency = 143 ± 14 Hz; Fig. 2c). Consistent with previous observations^34^,^35^, the individual ripple events lasted 37.7 ± 1 ms and occurred at 0.11 Hz (median value, n = 3 mice). During locomotion, 39–86% (interquartile range) of walking and running episodes were associated with theta oscillations, while 82–93% (interquartile range) of the theta oscillations recorded in the experiment occurred during locomotion episodes. Moreover, there were significant positive correlations between the animal’s speed and the theta oscillation power and frequency in 69% and 63% of cases, respectively (Fig. 2d, n = 5 mice, median slopes: 0.023 dB/cm.s^−1^ and 0.01 Hz/cm.s^−1^). Taken together, these data confirm the previous observations of the LIA and ripples during immobility and of theta oscillations during locomotion in head-fixed and freely behaving rodents^18^,^26^,^27^,^28^,^31^,^35^.

The behaviour was spontaneous, transitions from immobility to locomotion occurred as the mouse desired. To obtain reliable quantitative data, we recorded from five mice during first seven consecutive days of head restriction, with two 5-min daily periods of recording (Fig. 2e–k). The spontaneous behaviour of the mice alternated between immobility/flickering (median and interquartile range durations: 9.8 and 2.0–10.5 s, n = 845) and locomotion episodes (n = 915). The total distance travelled during locomotion (median and interquartile range: 107 and 15–114 cm) and the locomotion duration (12.1 and 3–15 s) had broad log-normal like distributions (Fig. 2e), whereas the locomotion speed (Fig. 2e, 7.5 and 4.4–9.8 cm s^−1^) was distributed around the median value. In line with previous observations of spontaneous behaviour in head-fixed mice^12^, the fluctuations in the locomotion parameters were broad, so we examined the source of this variability by analysing, first, the daily evolution of the median speed and speed stereotypy (Fig. 2f). The results of this analysis showed that the median speed exhibited by the animal increased significantly from day to day, reaching a plateau after 4 days of the spontaneous head-fixed behaviour. By contrast, the speed stereotypy improved significantly over time (Fig. 2f, bootstrap procedure, *P* < 0.01). Given that animal speed can be used to determine behavioural phases, we considered the distribution of each phase in the full dataset and found that most of the spontaneous behaviours comprised locomotion (53.7%), followed by immobility (32.4%) and flickering (13.5%; median values, Fig. 2g, n = 5 mice). We defined flickering as a transitional state associated with animal adaptation on the wheel and examined whether it could be used as an animal habituation index. Indeed, after comparing the evolution of the three behavioural patterns over several days, the flickering phase displayed a significant decrease (26.5%, Fig. 2h) thereby indicating that the fraction of time occupied by the flickering periods could indicate an animal’s habituation level.

Second, consistent with the typical observations on freely behaving rodents, there was significant variability in the head-fixed behaviours of individual mice, which persisted for days (Fig. 2h,i). In particular, the inter-individual variability was significantly higher than the intra-individual variability (Fig. 2i, Wilcoxon signed rank test, *P* < 0.05). To examine further whether the mice in our experimental paradigm displayed behaviour similar to freely moving mice, we analysed the dataset at a smaller time scale in response to changing environmental conditions, such as the switch in light exposure (Fig. 2j). In both the light and dark conditions, the probability of run epochs occurring was similar (Fig. 2j,k) and it decreased within the first 5 min of animal positioning in the apparatus (Wilcoxon signed rank test, *P* < 0.05, n = 5 mice; Fig. 2k), which may be related to the extinction of “exploratory” behaviour^36^ in head-fixed mice. These data indicate that mice fixed in our platform generate behaviours similar to that in freely moving animals, with expected inter- and intra-individual variability.

### Counting the steps walked

**Figure 3:**
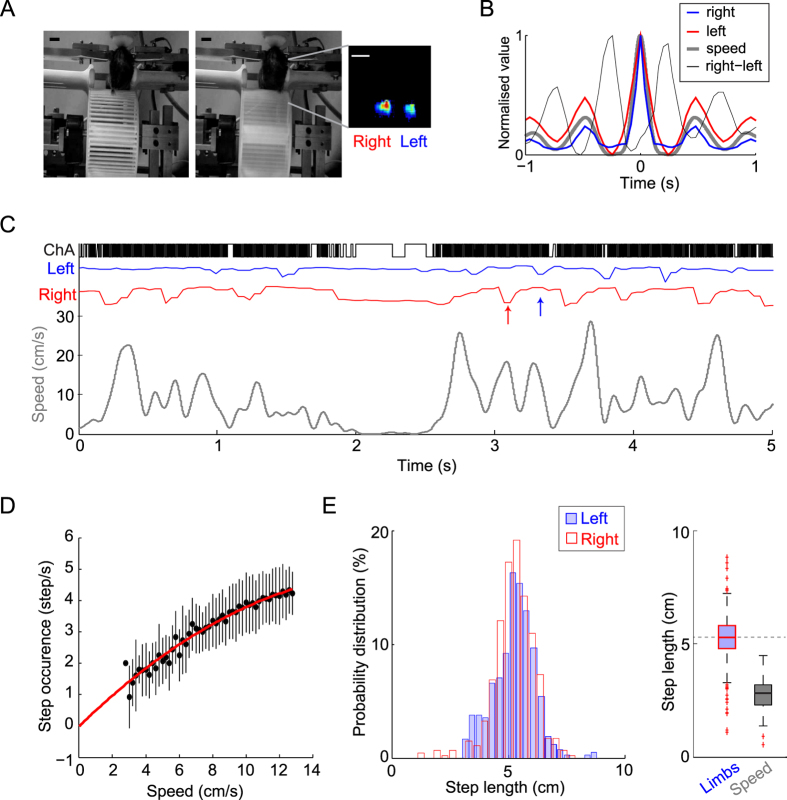
Counting the steps walked. (**A**) Illustration of mouse during a step, where the right then left fore-limb grabs the wheel bars. The heatmap shows principal component maps of the forelimb grabbing in a 4-min movie. Scale bar, 10 mm. (**B**) Autocorrelation functions for the right leg (blue), left leg (red), and instantaneous speed (grey, thick line), as well as the cross-correlations between the two legs (black). (**C**) Illustrations of channel A (black), left leg (blue), right leg (red), and speed (grey). Note the downward deflections of the leg signals (arrows) whereas the speed signal oscillates. (**D**) Relationship between the step occurrence and the median speed, with a red line indicating the least squares fit for the left leg. Pearson’s correlation coefficient: r = 0.985, p < 0.001. (**E**) Distribution histogram of the step length obtained from the right (blue) and left (red) legs (left) and boxplots (right) showing the distribution of the step length from both limbs (blue/red) and the distance between two cycles of the speed signal (gray).

As this behavioural platform can be potentially adapted to various experimental paradigms, we examined whether additional behavioural readouts could be obtained from the optical encoder signals acquired at a high temporal resolution. We noticed a slow frequency oscillation in the speed trace (Fig. 1d) and tested whether this rhythmic activity could be associated with the walking pace. We obtained video recordings of the animal’s steps from the front view and isolated the signal of forelimbs moving forwards (Fig. 3a,c). The autocorrelation analysis confirmed that the right and left forelegs oscillated together with the speed trace at the same rhythm. Moreover, cross-correlation analysis indicated that the left and right signals were in anti-phase (Fig. 3b). After temporal alignment (Fig. 3c), we found that the signal obtained from the encoder had an interesting pattern with an increasing density of fringe breaking, which reflected a wheel pooling after a right to left transition, and a decreasing density, which corresponded to slowing down of the foreleg followed by another left to right transition. To test whether the speed of the oscillations could predict steps, we detected steps from the video signals and looked at the correlation between the speed and the number of steps (Fig. 3d; Pearson’s correlation coefficient: r = 0.985, *P* < 0.001). As expected, this relationship intercepted at zero and was linear for the range of the video signal temporal resolution. The step length (Fig. 3e) exhibited a tight distribution (median: 5.0 ± 1.1 cm per step). In addition, we observed that the step length extracted from the speed signal (see methods) corresponded to the half of the limbs step size (2 limbs transitions for a complete step cycle).

### CA1 cellular activity during spontaneous behaviour

**Figure 4:**
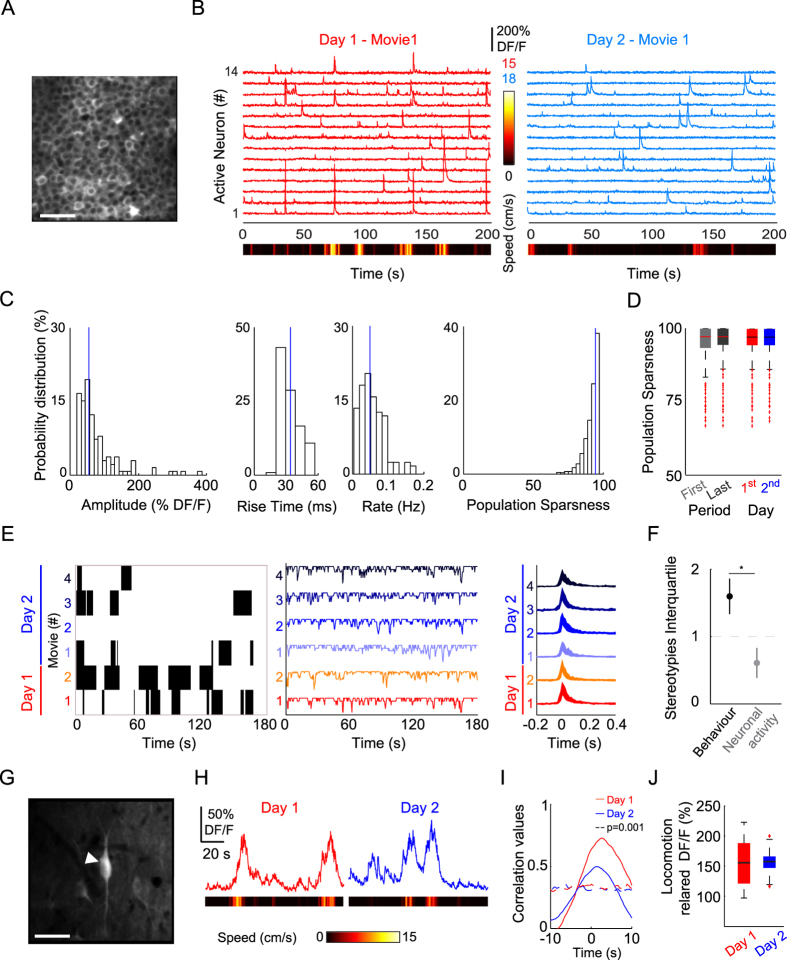
CA1 neuronal activity during spontaneous behaviour. (**A**) Median image at the level of the CA1 stratum pyramidale cell layer. Scale bar: 50 μm. (**B**) Plots showing the fluorescent traces from CA1 active neurons extracted from the first movies on day 1 (left, red) and day 2 (right, light blue). The heat map represents the magnitude of speed. (**C**) Histograms showing the distribution of the calcium transient amplitude, rise time, rate and population sparseness values. Medians (blue line) are superimposed. (**D**) Box plots showing the intra-movie (left) and inter-movie (right) variability. The distributions of the population sparseness for the first half (light grey) or last period (dark grey) of the movie and for the first (red) or second (blue) day did not differ significantly (*P* > 0.5, Wilcoxon signed rank test). (**E**) Locomotion epoch patterns (black, left), colour-coded traces representing the population sparseness (middle), and the population calcium transient interquartile range (right) were extracted from six movies over 2 days. (**F**) Quantification of the distribution of stereotypy for behaviour (black, n = 4) and neuronal activity (grey, n = 4) distributions. Data represent the mean ± standard deviation (*indicates *P* < 0.05, Wilcoxon signed rank test). (**G**) Median image of a stratum oriens CA1 GABAergic neuron. Scale bar: 50 μm. (**H**) Calcium traces of the same cell (arrow-head in (**G**) from day 1 (red, left) and day 2 (blue, right) during spontaneous locomotor behaviour. The heat map represents the magnitude of speed. (**I**) Plots showing the calcium to speed trace normalized covariance for day 1 (red) and day 2 (blue). Chance levels at *P* = 0.001 are shown as dashed lines (colour coded according to days). (**J**) Box plots showing the non-significant difference in locomotion-related DFF for day 1 (red) and day 2 (blue).

Given that spontaneous behaviour was associated with high intra-session and inter-session variability (Fig. 2i), we next examined the variability in the activity of CA1 neurons. We first recorded somatic Ca^2+^ transients from the CA1 pyramidal layer neurons that were targeted with the Ca^2+^-sensitive protein GCaMP6f through stereotaxic injection of AAV1.Syn.GCaMP6f.WPRE.SV40 in the CA1 area (Fig. 4a,b). Somatic Ca^2+^ transients had broad but log-normal-like amplitude distributions (Fig. 4c). At single cell level, the median values for Ca^2+^ transients rise time and rate reached 33.7 ms and 0.05 Hz, respectively (Fig. 4c). At population level, we first performed pairwise analysis to reveal the neuron-to-neuron correlations and found a very low level of correlation between individual cells (75^th^ of the pairwise correlation: 0.065). To better examine the population activity, the population sparseness was then computed and demonstrated a skewed distribution consistent with sparse coding (Fig. 4c). Using this metric, we found that despite fluctuations in behaviour, the intra-session and inter-session recruitment of CA1 neurons were similar (Fig. 4d). Next, given that animal behaviour was highly variable (Figs 2i and 4e), we performed a paired comparison of the behaviour and the neuronal activity distributions, which showed that the neuronal activity metrics had a significantly more stereotyped distribution compared with the behaviour metrics (Fig. 4f). Thus, despite the significant fluctuations in the spontaneous motor behaviour of mice, the CA1 pyramidal cell network exhibited a rather narrow repertoire of activities. In addition, we imaged GABAergic neurons in the CA1 stratum oriens area (Fig. 4g,h) and found that the activity of these cells also had the same relationship with behaviour on a daily basis (Fig. 4h), where the amplitude of the Ca^2+^ transients correlated well with the locomotion epochs (Fig. 4i,j). These results indicate the overall conservation of the CA1 network activity patterns over different time scales.

**Figure 5:**
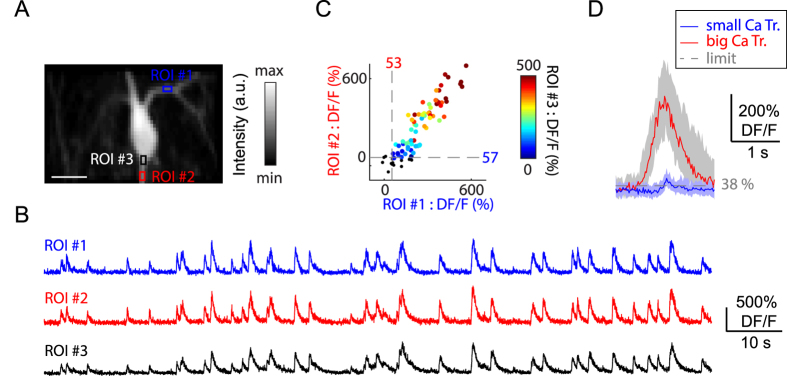
*In vivo* two photon imaging of interneurons proximal dendrites. (**A**) Average image of a VIP expressing CA1 stratum oriens neuron with dendritic ROIs (coloured squares) and soma border ROI (black), scale bar: 20 μm. (**B**) Calcium fluorescent trace for color-coded ROIs. (**C**) Scatter plot of Ca^2+^ transient peak amplitude for ROI #1 (x axis) and ROI #2 (y axis), the colour code corresponds to the value of the soma border ROI. Grey dashed lines indicate minimal detection levels. (**D**) Distribution for the smallest (n = 40) and biggest (n = 46) detected Ca^2+^ transients. Grey dashed line indicates median detection levels across 6 dendrites.

In order to estimate the range of detectable Ca^2+^ transients’ amplitudes in proximal dendrites and axonal boutons during spontaneous behaviour, we imaged these compartments on GABAergic neurons. To achieve a sparse population labelling of neurons, we choose an interneuron subtype that expresses vasoactive intestinal peptide (VIP)^37^, since these cells comprise a small subpopulation of CA1 interneurons and their recruitment during different behavioural states is unknown compared to other interneuron classes^24^,^27^,^28^,^31^. First, we imaged Ca^2+^ transients in the proximal dendrites compared to soma of the VIP-expressing cells targeted with AAV1.Syn.Flex.GCaMP6f.WPRE.SV40 in VIP-Cre mice (Fig. 5a,b). The amplitude of Ca^2+^ transients detected in proximal dendrites of VIP interneurons (<50 μm) was similar to that obtained at the soma border level (Pearson’s correlation coefficient: r = 0.93, p < 0.001; Fig. 5b,c). We examined the variability in the amplitude of the Ca^2+^ transients to determine a threshold Ca^2+^ signal that could be detected reliably in proximal dendrites. Using an adaptive algorithm (see methods), we found that the smallest Ca^2+^ transient detected by proximal dendrites should be at least 38% ∆F/F (median value, n = 6 dendrites, 3 neurons; Fig. 5d). The range of detectable amplitudes for dendritic Ca^2+^ transients (38 up to 562%; Fig. 5d) indicated that the proximal dendrites could experience a large repertoire of Ca^2+^ signals in behaving mice *in vivo* (likely evoked by different electrical events from single spikes to bursts of firing), with a potential impact on synaptic efficacy^38^,^39^.

### Lateral and axial stability

**Figure 6:**
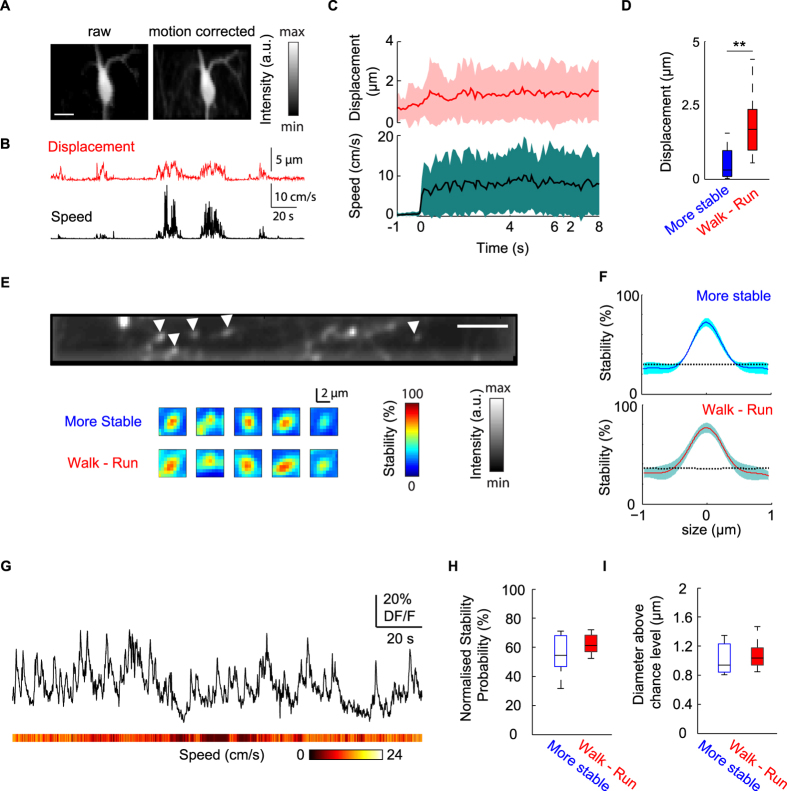
Lateral and axial stability during two-photon imaging. (**A**) Median images of the hippocampal CA1 stratum oriens area where GCaMP6f was expressed in a VIP interneuron without (left) and with (right) motion correction processing (scale bar, 20 μm). (**B**) Representative traces of motion related displacement (top, red) and speed (bottom, black) are plotted over a two minutes period. (**C**) Distribution of displacement (top, red) and speed (bottom, black) triggered by walk-run epoch initiation (n = 52 events, 7 movies) are plotted where line and filled area represent respectively median and interquartile range over an 8 seconds period. (**D**) Quantification of lateral motion across most stable (blue) and walk-run (red) groups (n = 7 movies, **P < 0.01, Wilcoxon test. Data analysis (**E**) Representative median image of GCaMP6f expressed in VIP-positive boutons (top, white arrowheads, scale bar: 10 μm). Cropped heat maps represent stability probability maps during more stable periods (middle) or walk-run epochs (bottom) for VIP-positive terminals shown on top. (**F**) Representative distribution of the stability profile (median ± interquartile range, n = 5 terminals) during stable periods (blue, top) and walk-run epochs (red, bottom). Black dashed lines indicate the spatial reshuffling median level. (**G**) Fluorescence signal over time of a single VIP bouton, heat map shows speed magnitude. (**H**) Box plots showing the distribution of the maximal amplitude of the normalized stability probability from more stable period (blue) or walk-run epochs (red) (n = 10 terminals, 3 movies, *P* > 0.1, Wilcoxon signed rank test). (**I)** Box plots showing the distribution of measured diameter of stable structure above chance level from more stable period (blue) or walk-run epochs (red) (n = 10 terminals, 3 movies, Wilcoxon signed rank test, *P* > 0.1).

Given that a good brain stability is required for imaging fine cellular structures, we examined the stability of our behavioural platform by analysing lateral and axial brain motions (Fig. 6a). Lateral motions were extracted by the brain motion artifact correction algorithm, which demonstrated the predominance of medio-rostrally oriented motion (data not shown). The magnitude of motion was tightly linked to locomotion onset (Fig. 6b,c) but was relatively low during run epochs (median: 1.8 μm, 75^th^: 2.5 μm). There was a statistically significant difference with a 5.6-fold increase in the motion magnitude between the most stable periods and walk-run epochs (Fig. 6d, n = 3 mice, 7 movies). Thus, we used only these two groups in the subsequent characterization of axial stability.

The axial stability was estimated based on the probability to keep in focus small cellular compartments such as the putative axonal boutons of VIP interneurons that innervate the CA1 stratum oriens/alveus^37^,^40^,^41^. To establish quantitative parameters for measuring stability, we computed a stability probability map to keep in focus the region of interest (ROI) containing boutons during locomotion epochs, and compared this map with a map computed from the period with the least lateral motion (Fig. 6d). To minimize the impact of signal variation during these two selected conditions, spatial reshuffling (Fig. 6e) was conducted for each selected image and the Ca^2+^ transients of a single VIP bouton could then be extracted (Fig. 6f). The peak of stability normalized for locomotion episodes (57–68%) and the most stable epochs (47–68%, interquartile range) did not differ significantly between the two states (n = 10, Wilcoxon signed rank test, *P* > 0.2; Fig. 6g,h). From the circular structure of boutons, the diameter above the 95th percentile in the reshuffled data indicated that there was no significant difference between the locomotion and stable state distributions (Fig. 6i; stable interquartile ranges: 0.85–1.24 μm; locomotion: 0.94–1.19 μm, n = 10; Wilcoxon signed rank test, *P* > 0.4). These results highlight the utility of our platform for imaging small axonal boutons during locomotion periods in the awake mouse.

## Discussion

Multimodal probing of neural circuits and circuit components with high spatial and temporal resolution in animals during behaviour is crucial for understanding how the brain shapes behaviour. Experiments relying on longitudinal recordings on head fixed animals performing controlled behavioural tasks can help to understand this relationship. We have developed a head fixation behavioural platform that has the ability to monitor distinct behavioural patterns, such as immobility, flickering, walking, and running, as well as imaging genetically defined neuronal populations and recording network oscillations in specific brain areas. The reliable tracking of the ongoing activity of an animal using an optical encoder allows users to precisely monitor rapid changes in the animal’s position, speed, and other locomotor patterns, including the incidence of walking steps. In particular, the optical encoder signal can be used to derive the step length, frequency and speed acceleration patterns. Such data are usually obtained using motion analysis techniques combined with high-speed video recordings of the behaviour^42^, or even more advanced four-axis robotic system (mouse stepper) equipped with leg-guidance linkages, motors and series of optical encoders that record the rotational position of the motors^43^,^44^. The latter, however, has a major advantage and application in both collecting hindlimb position data and guiding hindlimbs following the injury. Our platform equipped with a simple optical encoder provides for an easy solution for step counting and analyses, which can be performed simultaneously with electrophysiology and two-photon imaging, and does not require additional video footage and separate data processing. Such data can be useful in studies oriented on cellular and circuit mechanisms of the motor control and spatial navigation.

In addition, the stability of the platform allows Ca^2+^ events to be recorded in cell populations and fine subcellular structures, such as neuronal dendrites and axonal boutons, with minimal motion artifacts. The ability to record the electrophysiological signal from the distant area will enable one to interrogate functional neural ensembles connected through long-range projections. The platform thus enables high-throughput and high-precision optophysiological experimentation on the rodent brain *in vivo*.

Several studies described the cellular activities in behavioural and network states^12^,^14^,^18^,^28^,^30^,^31^,^32^,^33^. However, little is known about the progress of neuronal patterns in line with evolving spontaneous behaviour of mice under head fixation, which is important for standardization of chronic observations between different laboratories. In this study, we used a quantitative approach to examine the individual, inter-session, and intra-session changes in animal behaviour and neuronal activity from the third day of head fixation (Day 1 in this study). Our data revealed a high level of inter-individual and inter-session variability, thus indicating that chronic imaging experiments on head-fixed behaving mice may require a large number of animals for statistically solid conclusions to be drawn. The CA1 activity in our study was sparse and stereotyped, which can be explained by the absence of spatial tasks, reward or training paradigms, thus providing control observations for advanced optophysiological experiments^15^,^18^,^19^.

Our quantitative analysis of the stability of the brain for imaging small neuronal structures provided good estimates of chronic imaging limitations; however they should not be taken as absolute values because (1) brain motion depends on the success of surgical procedures and the quality of mouse training, (2) a signal threshold was used to compute the stability maps, and (3) the point spread function broadens the size of the scanned focal plane axially depending on the depth-induced scattering and microscope settings^45^. In addition, the variability of dendritic Ca^2+^ events may have been attributable to the three-dimensional (3D) geometrical structure of the dendritic branch and the associated challenge of keeping a long segment within the same focal plane. To compute the calcium dynamics from VIP-positive axonal terminals, we inferred their circular structure, which is a necessary manipulation when estimating the degree of stability for an axonal bouton. The values obtained were consistent with those reported for the VIP bouton size typical of type 3 interneuron-specific interneurons^37^,^41^, thus indicating the validity of this approach for quantification of the bouton Ca^2+^ events. A better stability in imaging subcellular structures could be achieved using a larger and thicker head plate; however, such tool may be too bulky and heavy, with a potential impact on animal post-surgery mobility and recovery. Thus, additional efforts may still be required towards optimization of such devices.

The main limitations of our head fixation platform, which should be considered as future areas for improvement, include: (1) the requirement for predetermining the head plate orientation during surgery; (2) the obvious functional clamping of vestibular system and head direction cells dynamics^46^,^47^ due to the head fixation itself; and (3) the limited postures available to the animal when the head is fixed. The animal habituation to the behavioural paradigm can be rapidly achieved through the handling procedure. Indeed, mice fixed in our platform exhibited voluntary locomotor behaviour with a progressive decrease in flickering (a putative learning index that indicates the optimal body posture) and gradually improved stereotypy in the median speed distribution, thereby indicating the sharpening and refinement of locomotor skills.

In summary, the simple head fixation platform developed in this study allows spontaneous neuronal activities to be recorded in parallel with network patterns and simple behavioural states. Importantly, the datasets obtained during spontaneous, “free-will” mouse behaviour in a simplified context can provide control observations for use in sensory-enriched experiments^18^,^19^, behavioural conditioning^24^, and more complex behavioural patterns in advanced virtual reality systems^14^,^15^,^30^. Thus, this simple flexible experimental platform should be a useful tool for the broad community of neuroscientists, and should allow investigating genetically defined neuronal circuits in the awake brain to provide fundamental new insights into brain computations *in vivo*.

## Materials and Methods

### Mice

Male adult wild-type C57BL6 or vasoactive intestinal peptide (VIP)-IRES-Cre (Jackson #010908) mice (n = 8, 25–35 g body weight) were used in the experiments. All animal experimentation was conducted in accordance with the Animal Protocol “Optical imaging and electrophysiological recordings in the mouse brain *in vivo*” (Protocol #2015097), which was approved by the Animal Protection Committee of Laval University in line with the guidelines of the Canadian Council on Animal Care. Mice were housed in standard conditions (12 h/12 h light/dark cycle with the light on at 07:00, one per cage, with water and food *ad libitum*) and handled before recording sessions to limit head restraint-associated stress.

### Experimental procedures

Stereotaxic injection of AAV1.Syn.GCaMP6f.WPRE.SV40 or AAV1.Syn.Flex.GCaMP6f.WPRE.SV40 (Penn Vector Core) (stock diluted 1:4 in phosphate-buffered saline, injection volume 100 nl) was performed in the CA1 hippocampus using the following coordinates: AP +2.4 mm, ML ±2.4 mm, DV −1.3 mm. After recovery for 4–6 days, a 3-day water restriction schedule of 1.5 ml day^−1^ was applied. At 7–10 days after viral injection, mice were anaesthetized deeply with a ketamine–xylasine mixture (100–10 mg kg^−1^), and fixed in a stereotaxic frame. A glass-bottomed cannula was inserted on top of the dorsal hippocampus after the cortex aspiration, and secured with Kwik-Sil at the tissue interface and Superbond at the skull level. A single tungsten electrode for local field potential (LFP) recordings was implanted in the contralateral CA1 hippocampus^35^. The head plate was oriented medio-laterally at 7–13° using a four-axis micromanipulator (MX10L, Siskiyou) and fixed with several layers of Superbond and dental cement. Mice were allowed to recover for several days with postoperative pain killer treatment for 3 consecutive days (buprenorphine, 0.1 mg kg^−1^; 48 h).

Behavioural habituation involved progressive handling by the experimenter for ~5–15 min twice per day, with the animal fixation in the apparatus starting from the third day. The aluminium head plate (0.92 g) was secured using a piton placed at the end of the bilateral holding arms (Supplementary Fig. 1c). The upper parts of the holding system were lowered using hex-ended screws.

### Experimental set-up

The head fixation platform was designed using SolidWorks (2015, premium) (all files are available upon request) and produced by a precise computer-assisted system from 316L stainless steel metal. The wheel and wall were 3D-printed from white plastic material. The head fixation platform was fixed on an XY movable platform (Scientifica) and the gross axial setting was adjusted using axial jacks (Thorlabs). An optical quadrature encoder (HEDS-5645#A06, Avago Technology) was aligned precisely and connected to an electronic custom-designed interface (Fig. 1c,d), with a 12-V power supply. Three channels were connected to a digitizer (DigiData 1440a, Axon Instruments). Channel I generated a 5-V inflection each turn (241.9 mm), and channels A and B generated 500 square inflections per turn, which were offset from 0.25 pitch to capture the direction of motion^48^. Recording and reference electrodes were connected to an amplifier (AM Systems). The LFP signal was amplified 1000 times and digitized simultaneously with the optical encoder channels and imaging trigger at a sampling frequency of 10 kHz (minimal value for optical encoder signals reliability) using Axoscope software (v10.5, Axon Instruments).

For spontaneous behaviour and LFP recordings without imaging, the mice were head fixed and the electrodes were connected. Two 5-min long recording sessions were acquired, with the first in light and the second in dark conditions on consecutive days.

Imaging was performed using a Leica SP5 TCS two-photon system and a Ti:sapphire femtosecond laser (Chameleon Ultra II, Coherent), which was mode-locked at 900 nm. A long-range water-immersion 25× objective (0.95 NA, 2.5 mm working distance; Leica) was used for excitation and light collection to external photomultiplier tubes (Leica) at 12 bits. Image series were acquired at axial resolutions of 0.3–2 μm pixel^−1^ and temporal resolutions of 8–39 images s^−1^. The imaging sessions lasted up to 3 h, after which the mouse was placed back in its home cage. The locomotion wheel was then cleaned with tap water.

### Data analysis

#### Spontaneous behaviour

The signals and image series were analysed using custom-made routines written in Matlab (MathWorks). To extract the animal position and speed, an algorithm derived from Patrascoiu *et al*.^48^ was adapted and validated using a calibration provided by a speed controller engine (OmniDrill35, WPI). A linear relationship was obtained over a wide range of speed (0–120 cm s^−1^), so this algorithm appeared to be highly reliable (Fig. 1e). The median locomotion speed in each turn was determined as:


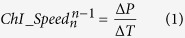


where *P* is the spatial position, *n* is the *n*^th^ detected channel I peak and *T* is the temporal position.

Three behavioural phases were identified: locomotion, flickering, and immobility. Locomotion epochs were defined as the periods when the instantaneous speed was higher than 2 cm s^−1^ for a minimal distance of 2 cm, thereby pooling together the walking and running periods. A transitional stage associated with the animal adaptation on the wheel and brief random movements translated into flickering, which was detected as periods when the speed was above 0.25 cm s^−1^ but below the locomotion threshold. Immobility periods were defined as the times without wheel rotation. These phases were translated into logic vector templates for subsequent analysis. The speed stereotypy for an experimental session was calculated as:


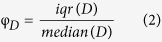


where *φ* is the stereotypy value, *D* is the distribution, *iqr* is the interquartile, with a value higher than one indicating a broad distribution and a value closer to zero showing a stereotyped distribution. Such method gives several advantages: (i) it allows to compare different distributions (normal, log-normal); (ii) it reduces outliers’ effect; (iii) it does not take into account absolute values.

#### Inter- and intra-individual variability comparisons

To compare the probability of variability in locomotion within the same and between different animals in each session, the following equation was applied:


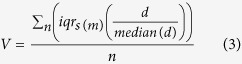


where *V* is the variability for a given condition (intra- or inter-individual), *d* is the distribution of the locomotion probability in a session *s, n* is the sample size of the condition (intra-individual: *n* = 7 days; inter-individual: *n* = 5 mice), and *m* is the sample size within the condition (intra-individual: *m* = 5 mice; inter-individual: *m* = 7 days). Thus, the spread of stereotyped locomotion probability by each mouse across days or for a day across all mice was analysed.

#### Relationship between speed and behaviour

A mini video camera (25 Hz temporal resolution, Sony) was used for video monitoring of animals in some experiments. The principal component analysis was applied at the level of the forelegs to derive the motion signals from the movie files. The frequency of steps was obtained from the number of left leg signal troughs per second. Despite the low resolution of the video acquired, the relationship was close to linear (Fig. 3d). To extract step size, peaks were detected in the speed signal and the derivative of their position was used to obtain a distance. Limbs are anti-correlated so the speed signal oscillated twice as fast as the limbs, thereby resulting in a twofold smaller step size for speed compared with the step size for limbs (Fig. 3e).

#### Hippocampal network states

For ripple detection, LFP traces were first band-pass filtered (100–250 Hz, fourth-order Butterworth filter) and the candidate ripple events were selected semi-automatically based on their power (≥3 SDs of the power of the full trace) with a minimal number of 4 ripple cycles. Visual validation of the raw data was performed for each preselected candidate event to remove false-positive artifact events. Ripple frequency (F) and occurrence (O) were obtained using equations 4 and 5:


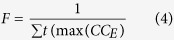



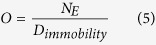


where *t* is the temporal position, *CC* is the cross-correlation of event segment *E* (100 ms), *N* is the number of event and *D* is the duration.

Theta oscillations were isolated after band-pass filtering a raw signal (5–15 Hz, fourth-order Butterworth filter), and the instantaneous frequency was obtained by computing the weighted frequency of a Morlet wavelet-based spectrogram. Theta power was integrated in a 1-s temporal window. For speed/theta correlations analysis, the animal speed was binned every 0.1 cm s^−1^.

### Image processing: brain motion

Series of movies were processed using a motion correction algorithm^12^, which detects the XY drift in each plane based on the cross-correlation with a reference image selected from a period that is free of motion and activity. Locomotion and immobility templates were down-sampled according to the temporal resolution of the Ca^2+^ imaging movie to segregate behavioural states. As behavioural immobility does not translate into brain immobility, a template called “more stable” was computed by selecting the immobility periods where the brain motions in all directions were below the average motion-vector length.

To estimate the axial stability, we computed a stability map for locomotion and the “more stable” conditions for VIP-positive axonal boutons in the CA1 stratum oriens. First, a reference image was generated from the median multiplied by the SD of the raw signal (8 out of 10 terminals) in order to obtain the best contrast image with terminals in focal plane. A mask was obtained after binarizing the region of interest with a threshold equal to the 90^th^ percentile of the intensity distribution for each frame in a given template. Then, for each pixel, the percentage of frames (value = 1) was estimated and 2D map of the percentage plotted. For statistical purpose, spatial reshuffling of the raw signal was computed for each frame by performing permutations of the pixels within their own frames for 1000 surrogates. The 95^th^ percentile of the surrogate stability map was used as a statistical threshold for quantifications.

The changes in fluorescence (*F*) associated with Ca^2+^ elevations were obtained by averaging the pixels inside the thresholded structure over time and expressed as:


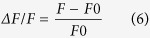


where *F*0 is the fluorescence baseline obtained by taking the main mode of the calcium trace distribution in the 5^th^–10^th^ range.

### Image processing: neuronal activity

Movies from the same field of view in the CA1 pyramidal cell layer were processed to extract active cells based on the presence of Ca^2+^ transients in the fluorescence traces^12^,^19^. Cell Ca^2+^ transients were extracted using a threshold method (mean + 2SD) based on the first derivative of the trace and a skewness above 1, which excluded minor or complex Ca^2+^ signals. The Ca^2+^ transient peak amplitude was defined as the median value of the cell Ca^2+^ transients normalized by the median value of the baseline period. The population Ca^2+^ transient was obtained as the median of the cell Ca^2+^ transient. The pairwise correlation was done on cell fluorescence traces (eq. 6) using the Cross-covariance function estimates (Matlab) normalised by signals standard deviations. To characterize the intra- and inter-session variability, the population sparseness was computed with a 500-ms time bin^49^. Briefly, we calculated how cells are active together. First, we set a threshold value for each cell using a threshold adaptive algorithm. This algorithm computes the skewness of the data at each cycle of a loop in which highest data points are progressively withdrawn. The threshold corresponds to the standard deviation of the data when asymmetry is null (skewness equal zero). Any neural responses whose magnitudes are larger than the threshold are considered to be “on”, and responses smaller than the threshold are considered “off”. The population activity sparseness is the distribution of the fraction of cells that are off per 500 ms bin: so the higher is the sparseness, the lower is the coactivation. To compare behaviour and the stability of activity, the stereotypies of different parameters (for behaviour: speed, distance, duration of locomotion and of immobility periods; for cell activity: Ca^2+^ transient rise time, amplitude, and frequency; and population sparseness) were computed.

For segmentation of interneurons, a reference image (median, standard deviation, or skewness projection) was used. The fluorescence traces were processed based on the normalized cross-covariance with animal speed using a 10-s window. The significance level was obtained using 5000 surrogates and a 99^th^ threshold. Locomotion-related Ca^2+^ signals were obtained as the Ca^2+^ signals that occurred during locomotion epochs. For the analysis of dendritic Ca^2+^ transients, an average image was selected from the locomotion period. Next, the ROIs (size: 15–20 μm^2^) were defined manually with comparable baseline fluorescence intensities between the soma border and dendritic proximal branches. Raw Ca^2+^ fluorescence traces were normalized by the median value of baseline period (eq. 6). The minimal detection threshold was determined using an adaptive algorithm with skewness equal to zero (see above). To display small and large transients, Ca^2+^ transients of the 1^st^ to 15^th^ peak amplitude percentiles and of the 85^th^ to 100^th^ percentiles were pooled, respectively.

### Statistical analyses

Dependent variables were tested using the Wilcoxon rank-sum test and significant differences were accepted at *P* < 0.05, unless indicated otherwise. To test the significance of the changes across days, a bootstrap procedure was performed by comparing the least squares fit linear slope of the real values with 10000 similar least squares fit linear slopes obtained from the surrogate distributions by full dataset reshuffling with random permutations. Changes were considered to differ significantly if the slope value was greater than the 95th percentile or lower than the 5th percentile of the surrogate slopes for positive and negative changes, respectively. Pearson correlations were performed using corrcoef Matlab function, which provides *R* value effect size and a *P* value as an output of the null hypothesis statistical test.

## Additional Information

**How to cite this article:** Villette, V. *et al*. Simple platform for chronic imaging of hippocampal activity during spontaneous behaviour in an awake mouse. *Sci. Rep.* **7**, 43388; doi: 10.1038/srep43388 (2017).

**Publisher's note:** Springer Nature remains neutral with regard to jurisdictional claims in published maps and institutional affiliations.

## Supplementary Material

Supplementary Figure 1
