# Supplementary material for: Simple platform for chronic imaging of hippocampal activity during spontaneous behaviour in an awake mouse
Source: Sci Rep. 2017 Feb 27;7:43388. doi: 10.1038/srep43388 (PMC5327464; doi:10.1038/srep43388)
Supplement: Supplementary Figure 1 [file srep43388-s1.pdf]

Simple platform for chronic imaging of hippocampal activity during spontaneous behaviour in an awake mouse

Vincent Villette, Mathieu Levesque, Amine Miled, Benoit Gosselin, Lisa Topolnik

Supplementary Figure 1. Head fixation device design

A. Design of the installed platform on the XY motor-driven platform under the microscope. Scale bar represents 5 cm. B. Detail of the head fixation holder in the closed position. C. Head plate released in the open holder position. Note that the red pin (*) facilitates repositioning of the head plate position. D. Free floating of the wheel holder system due to the unfixed lateral bars, where the green lines indicate the sliding area. Note the double axis of the wheel together with the ball bearing (orange) to provide the least friction during rotations. E. Shock absorber comprising a soft wheel (bar diameter: 2 mm), and the free-floating wheel holder system sitting on springs. Note that the optical encoder is shown in black.

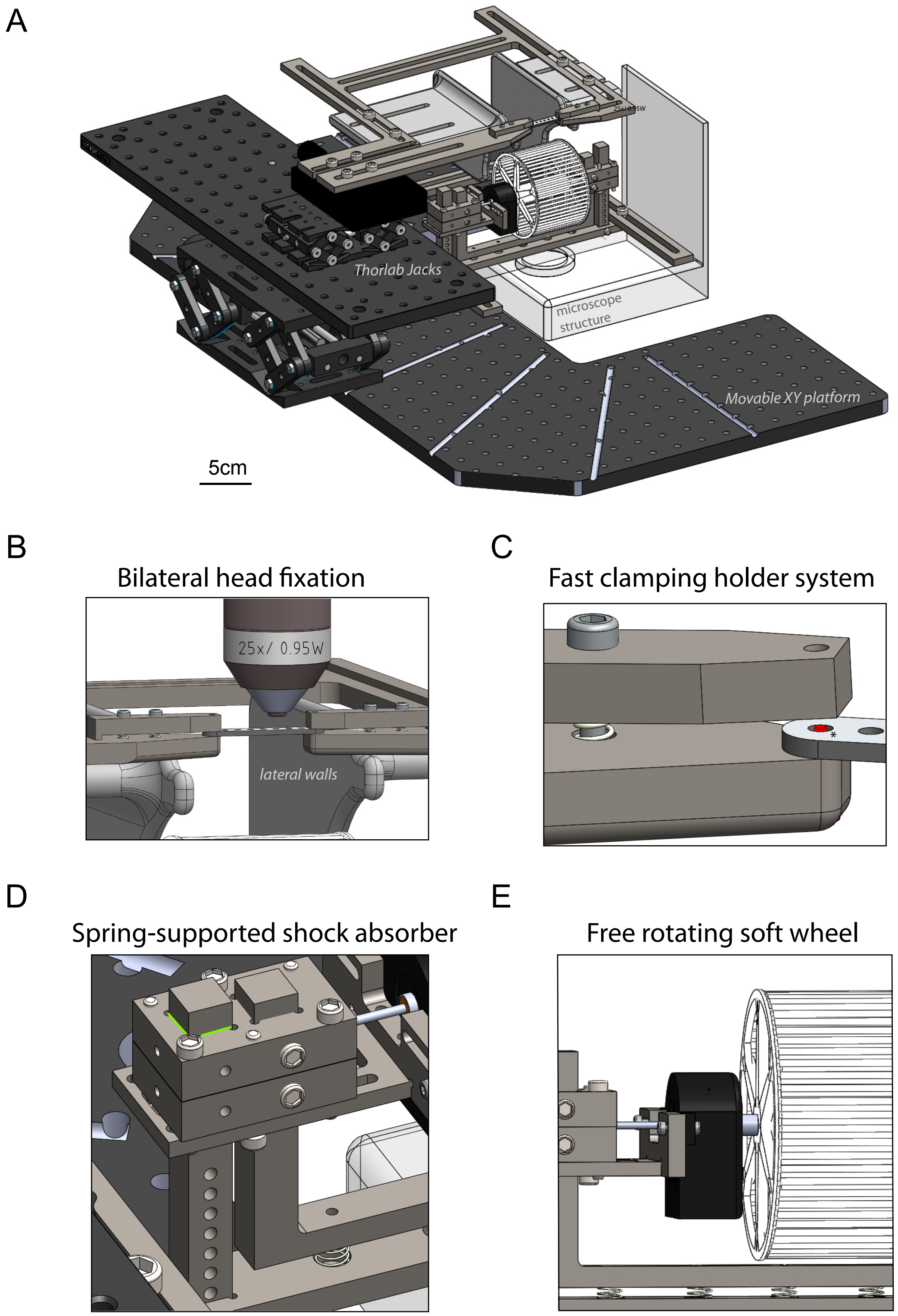

Supplementary Figure 1
